# {[Na1(μ-H_2_O)Na2]_2_[(C_2_O_4_)_2_Cr(μ-OH)_2_Cr(C_2_O_4_)_2_]·H_2_O}_*n*_, a novel hydrated form

**DOI:** 10.1107/S1600536810023986

**Published:** 2010-07-21

**Authors:** Michel M. Bélombé, Justin Nenwa, Jean Ngouné, Eleuterio Álvarez, Agustín Galindo

**Affiliations:** a Department of Inorganic Chemistry, Faculty of Science, POB 812, University of Yaounde I, Cameroon; b Department of Chemistry, Faculty of Science, University of Dschang, POB 67, Dschang, Cameroon; c Instituto de Investigaciones Químicas, CSIC, Universidad de Sevilla, Avda. Américo Vespucio 49, 41092 Sevilla, Spain; d Departamento de Química Inorgánica, Universidad de Sevilla, Aptdo 1203, 41071 Sevilla, Spain

## Abstract

The unit cell of the title compound, poly[[μ-aqua-μ-hydroxido-di-μ-oxalato-chromium(III)disodium] monohydrate], {[CrNa_2_(C_2_O_4_)_2_(OH)(H_2_O)]·H_2_O}_*n*_, contains four [Na1(μ-H_2_O)Na2][(C_2_O_4_)_2_Cr(μ-OH)·H_2_O] formula units, each of which consists of two crystallographically independent Na^+^ sites (bridged by one aqua ligand), one half of a centrosymmetric di-μ-hydroxido-bis­[*cis*-bis­(oxalato)chromate(III)] dimer, [(C_2_O_4_)_2_Cr(μ-OH)_2_Cr(C_2_O_4_)_2_]^4−^, and one uncoordin­ated water mol­ecule. The structure is best described as a coordination polymer in which the three-dimensional lattice framework is realized by the inter­connection of the metallic atoms *via* the O atoms of the aqua, hydroxide and oxalate ligands. One Na atom is hepta­coordinated by one water, one hydroxide and five oxalate O atoms, whilst the other is penta­coordinated by one water and four oxalate O atoms. The coordination around the Cr^3+^ sites is pseudo-octa­hedral, involving four aqua and two hydroxide O atoms. Adjacent Na atoms are separated by 3.593 (2) Å, whereas the intra­dimer Cr⋯Cr spacing is 2.978 (1) Å. The crystal structure is consolidated by extended relatively weak O—H⋯O hydrogen bonding with O⋯O distances ranging from 2.808 (4) to 3.276 (5) Å.

## Related literature

For general background, see: Ferreira *et al.* (2001 ▶); Köse *et al.* (2009 ▶). For a related structure with a different number of water molecules, see: Scaringe *et al.* (1977 ▶).
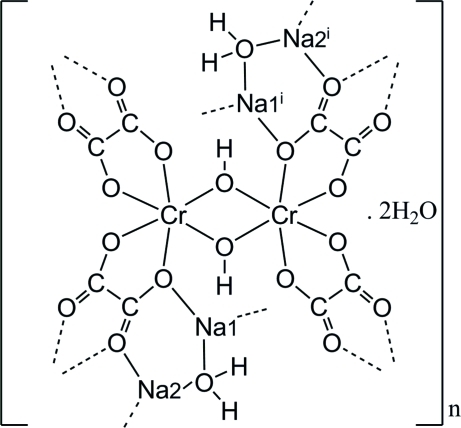


## Experimental

### 

#### Crystal data

[CrNa_2_(C_2_O_4_)_2_(OH)(H_2_O)]·H_2_O
*M* *_r_* = 327.06
Monoclinic, 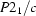

*a* = 9.4776 (10) Å
*b* = 8.603 (1) Å
*c* = 12.5353 (14) Å
β = 102.503 (2)°
*V* = 997.84 (19) Å^3^
*Z* = 4
Mo *K*α radiation
μ = 1.29 mm^−1^
*T* = 173 K
0.60 × 0.12 × 0.10 mm

#### Data collection

Bruker–Nonius X8 Kappa APEXII CCD area-detector diffractometer
Absorption correction: multi-scan (*SADABS*; Bruker 2005 ▶) *T*_min_ = 0.832, *T*_max_ = 0.882
13658 measured reflections
2018 independent reflections
1625 reflections with *I* > 2σ(*I*)
*R*_int_ = 0.037

#### Refinement

*R*[*F* ^2^ > 2σ(*F* ^2^)] = 0.044
*wR*(*F* ^2^) = 0.133
*S* = 1.14
2018 reflections
175 parameters
6 restraints
H atoms treated by a mixture of independent and constrained refinement
Δρ_max_ = 0.85 e Å^−3^
Δρ_min_ = −0.80 e Å^−3^

### 

Data collection: *APEX2* (Bruker, 2005 ▶); cell refinement: *SAINT* (Bruker, 2005 ▶); data reduction: *SAINT*; program(s) used to solve structure: *SIR2002* (Burla *et al.*, 2003 ▶); program(s) used to refine structure: *SHELXL97* (Sheldrick, 2008 ▶); molecular graphics: *SHELXTL* (Sheldrick, 2008 ▶); software used to prepare material for publication: *SHELXTL*.

## Supplementary Material

Crystal structure: contains datablocks I, global. DOI: 10.1107/S1600536810023986/pb2030sup1.cif

Structure factors: contains datablocks I. DOI: 10.1107/S1600536810023986/pb2030Isup2.hkl

Additional supplementary materials:  crystallographic information; 3D view; checkCIF report

## Figures and Tables

**Table 1:** Hydrogen-bond geometry (Å, °)

*D*—H⋯*A*	*D*—H	H⋯*A*	*D*⋯*A*	*D*—H⋯*A*
O11—H11*B*⋯O9^i^	0.88 (2)	2.23 (2)	3.080 (5)	163 (5)
O11—H11*A*⋯O6	0.88 (4)	2.53 (4)	3.144 (5)	128 (4)
O11—H11*A*⋯O3	0.88 (4)	2.25 (4)	2.949 (4)	137 (4)
O10—H10*B*⋯O5^ii^	0.88 (4)	2.36 (4)	3.123 (4)	144 (5)
O10—H10*A*⋯O5^iii^	0.91 (2)	2.02 (2)	2.922 (5)	179 (4)
O1—H1⋯O11^iv^	0.85	2.01	2.808 (4)	156

Symmetry codes: (i) 
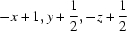
; (ii) 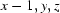; (iii) 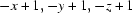; (iv) 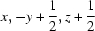.

**Fig. 1.**
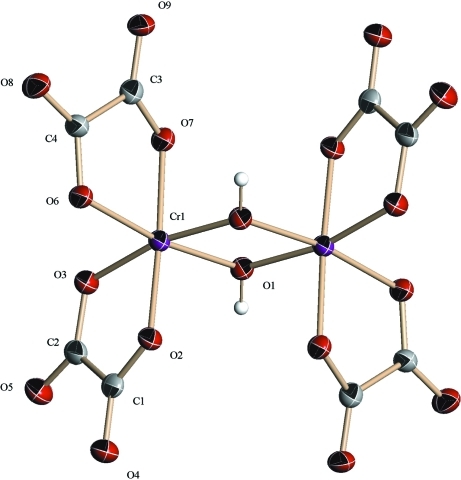
ORTEP drawing of the anionic dimer, [(C2O4)2Cr(µ-OH)2Cr(C2O4)2]4-, in the title compound with atom labeling and numbering. Displacement ellipsoids are drawn at the 50% probability level.

**Fig. 2.**
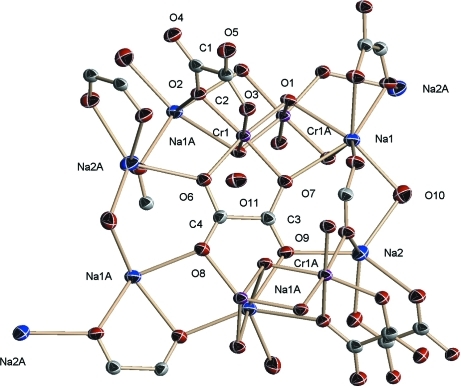
Larger portion of the structure of (I) projected onto the (-110) crystallographic plane, highlighting the nonmolecular coordinative polymerization

## supplementary crystallographic information

### Comment

Scaringe *et al.* (1977) have previously studied the system Na_4_[Cr(ox)_2_OH]_2_.6H_2_O (ox = C_2_O_4_^2-^), paying special attention to its structural-magnetic correlations. We report herein compound (I) as a novel hydrated form of this system, which involves a different number of water molecules and exhibits a different mode of coordinative polymerization.

Fig. 1 depicts the centrosymmetric anionic dimer, [(C_2_O_4_)_2_Cr(µ-OH)_2_Cr(C_2_O_4_)_2_]^4-^, which constitutes the main structural motif of (I). It is virtually identical to the motif reported previously (Scaringe *et al.*,1977), as revealed by the closely comparable geometric parameters (Table 1). A larger portion of the structure of (I) is shown in Fig. 2, highlighting heptacoordination of Na1, pentacoordination of Na2 by O atoms, and the bridging of these metallic sites by an aqua ligand (O10). The oxygen atom, O11, is seen to be part of a water molecule of crystallization.

Beyond their identical *P*2_1_/c space group, the two structures present a number of differing features. The crystal data of (I) compare with the reported values (in square brackets) as follows: *a* = 9.478 (1) [19.530 (12)] Å; *b* = 8.603 (1) [9.860 (7)] Å; *c* = 12.535 (1) [12.657 (10)] Å; *β*= 102.50 [106.93]°; *R*_final_ = 4.4 [6.1] %; *Z* = 4 [4]; *D_x_* = 2.177 [1.966] Mgm^3-^; *T*(K) = 173 [293]. The asymmetric unit of the reported structure contains six H_2_O molecules and four crystallographically independent Na^+^ sites, whereby Na1 and Na3 are bridged by the aqua oxygen atom, WO(2), and Na2 and Na4 by the aqua oxygen atom, WO(1), with each Na^+^ site assuming coordination number 6. The asymmetric unit of (I), by contrast, contains four Na^+^ ions (located at two crystallographically independent sites) and four H_2_O molecules. Note, furthermore, that coordination numbers 5 and 6 by O atoms have been frequently reported in the literature (Scaringe *et al.*, 1977; Ferreira *et al.*, 2001; Köse *et al.*, 2009), but coordination number 7, if any, is rather scarce for Na^+^. Like in the reported material, the bulk structure of (I) is consolidated by O—H···O bridgings which are, however, stronger in the former than in the latter case (Table 2).

Preliminary observations from our laboratory promisingly suggest that a well conceived and systematically conducted preparative procedure may be applied generally to fabricate a whole range of homologous magnetic materials, provided appropriate paramagnetic transition metal centers are involved.

### Experimental

Go In an initial attempt to prepare the targeted material of empirical formula [Ba_2_(H_2_O)_6_][Cr_2_(OH)_2_(C_2_O_4_)_4_].H_2_O, compound (I), instead, was obtained as follows: Cr(NO_3_)_3_.9H_2_O (4.0 g, 10 mmol, Riedel-de Haën, pure) was dissolved at room temperature in H_2_O (150 ml) and filtered. H_2_C_2_O_4_.2H_2_O (2.6 g, 20 mmol, Riedel-de Haën, 99.5–100.5%) was dissolved therein. This solution was added dropwise to a suspension of Na_2_CO_3_ (1.6 g, 15 mmol, Riedel-de Haën, 99%) and Ba(OH)_2_.8H_2_O (3.2 g, 10 mmol, Merck, p.a.) in 200 ml of water, and stirred overnight at *ca* 70 °C. The violet mass that had formed was filtered off, washed twice with 15 ml H_2_O and dried between filter papers. A second crop of material was obtained by concentrating the mother liquor to a volume of *ca* 20 ml, washing the material twice with 5 ml H_2_O and drying it as above. Recrystallization from oversaturated aqueous solution yielded prismatic violet crystals appropriate for X-ray diffractions.

### Refinement

The water hydrogen atoms were located from a difference Fourier map and refined isotropically, with the O–H distance restrained to 0.88 (4) Å, *U*iso = 1.5*U*eq (O). Other H atoms, for the OH group, were constrained to ideal geometries, with 0.85 Å.

### Crystal data

**Table:**

[CrNa_2_(C_2_O_4_)_2_(OH)(H_2_O)]·H_2_O	*F*(000) = 652
*M**_r_* = 327.06	*D*_x_ = 2.177 Mg m^−^^3^
Monoclinic, *P*2_1_/*c*	Mo *K*α radiation, λ = 0.71073 Å
Hall symbol: -P 2ybc	Cell parameters from 6874 reflections
*a* = 9.4776 (10) Å	θ = 2.2–26.1°
*b* = 8.603 (1) Å	µ = 1.29 mm^−^^1^
*c* = 12.5353 (14) Å	*T* = 173 K
β = 102.503 (2)°	Prism, violet
*V* = 997.84 (19) Å^3^	0.60 × 0.12 × 0.10 mm
*Z* = 4	

### Data collection

**Table:**

Bruker–Nonius X8 Kappa APEXII CCD area-detector diffractometer	2018 independent reflections
Radiation source: fine-focus sealed tube	1625 reflections with *I* > 2σ(*I*)
graphite	*R*_int_ = 0.037
Detector resolution: 8.26 pixels mm^-1^	θ_max_ = 26.4°, θ_min_ = 2.2°
phi and ω scans with narrow frames	*h* = −11→11
Absorption correction: multi-scan (*APEX2*; Bruker 2005)	*k* = −10→10
*T*_min_ = 0.832, *T*_max_ = 0.882	*l* = −15→15
13658 measured reflections	

### Refinement

**Table:**

Refinement on *F*^2^	Primary atom site location: structure-invariant direct methods
Least-squares matrix: full	Secondary atom site location: difference Fourier map
*R*[*F*^2^ > 2σ(*F*^2^)] = 0.044	Hydrogen site location: inferred from neighbouring sites
*wR*(*F*^2^) = 0.133	H atoms treated by a mixture of independent and constrained refinement
*S* = 1.14	*w* = 1/[σ^2^(*F*_o_^2^) + (0.054*P*)^2^ + 4.054*P*] where *P* = (*F*_o_^2^ + 2*F*_c_^2^)/3
2018 reflections	(Δ/σ)_max_ < 0.001
175 parameters	Δρ_max_ = 0.85 e Å^−^^3^
6 restraints	Δρ_min_ = −0.80 e Å^−^^3^

### Special details

**Table:**

Geometry. All e.s.d.'s (except the e.s.d. in the dihedral angle between two l.s. planes) are estimated using the full covariance matrix. The cell e.s.d.'s are taken into account individually in the estimation of e.s.d.'s in distances, angles and torsion angles; correlations between e.s.d.'s in cell parameters are only used when they are defined by crystal symmetry. An approximate (isotropic) treatment of cell e.s.d.'s is used for estimating e.s.d.'s involving l.s. planes.
Refinement. Refinement of *F*^2^ against ALL reflections. The weighted *R*-factor *wR* and goodness of fit *S* are based on *F*^2^, conventional *R*-factors *R* are based on *F*, with *F* set to zero for negative *F*^2^. The threshold expression of *F*^2^ > σ(*F*^2^) is used only for calculating *R*-factors(gt) *etc*. and is not relevant to the choice of reflections for refinement. *R*-factors based on *F*^2^ are statistically about twice as large as those based on *F*, and *R*- factors based on ALL data will be even larger.

### Fractional atomic coordinates and isotropic or equivalent isotropic displacement parameters (Å^2^)

**Table:**

	*x*	*y*	*z*	*U*_iso_*/*U*_eq_	
Cr1	0.60366 (7)	0.06037 (8)	0.43610 (5)	0.0210 (2)	
Na1	0.36188 (17)	0.3337 (2)	0.48733 (13)	0.0265 (4)	
Na2	0.14538 (19)	0.3470 (2)	0.21413 (15)	0.0327 (4)	
O1	0.5272 (3)	0.1171 (4)	0.5646 (2)	0.0237 (6)	
H1	0.5819	0.1507	0.6229	0.028*	
O2	0.7636 (3)	−0.0653 (3)	0.5189 (2)	0.0231 (6)	
O3	0.7506 (3)	0.2268 (4)	0.4659 (2)	0.0249 (6)	
O4	0.9836 (3)	−0.0410 (4)	0.6268 (2)	0.0298 (7)	
O5	0.9674 (3)	0.2755 (4)	0.5739 (3)	0.0313 (7)	
O6	0.6501 (3)	0.0117 (4)	0.2928 (2)	0.0249 (6)	
O7	0.4467 (3)	0.1816 (4)	0.3427 (2)	0.0242 (6)	
O8	0.5513 (3)	0.0306 (4)	0.1139 (2)	0.0281 (7)	
O9	0.3141 (3)	0.1684 (4)	0.1719 (2)	0.0277 (7)	
C1	0.8758 (4)	0.0129 (5)	0.5650 (3)	0.0241 (9)	
C2	0.8674 (4)	0.1875 (6)	0.5347 (3)	0.0256 (9)	
C3	0.4236 (4)	0.1425 (5)	0.2411 (3)	0.0230 (8)	
C4	0.5518 (4)	0.0536 (5)	0.2099 (3)	0.0222 (8)	
O10	0.1206 (3)	0.4049 (4)	0.3955 (3)	0.0344 (8)	
H10A	0.094 (5)	0.504 (2)	0.405 (5)	0.051*	
H10B	0.058 (5)	0.345 (4)	0.419 (4)	0.051*	
O11	0.7568 (4)	0.3460 (4)	0.2464 (3)	0.0370 (8)	
H11A	0.777 (6)	0.271 (4)	0.295 (3)	0.055*	
H11B	0.756 (6)	0.438 (3)	0.277 (4)	0.055*	

### Atomic displacement parameters (Å^2^)

**Table:**

	*U*^11^	*U*^22^	*U*^33^	*U*^12^	*U*^13^	*U*^23^
Cr1	0.0155 (3)	0.0329 (4)	0.0148 (3)	0.0002 (3)	0.0036 (2)	0.0002 (3)
Na1	0.0221 (8)	0.0386 (10)	0.0185 (8)	0.0001 (7)	0.0035 (6)	0.0001 (7)
Na2	0.0212 (9)	0.0438 (11)	0.0309 (9)	0.0028 (8)	0.0005 (7)	−0.0031 (8)
O1	0.0202 (14)	0.0366 (17)	0.0142 (13)	−0.0032 (13)	0.0033 (10)	−0.0020 (12)
O2	0.0176 (13)	0.0314 (16)	0.0195 (14)	−0.0025 (12)	0.0022 (11)	−0.0005 (12)
O3	0.0201 (14)	0.0319 (16)	0.0220 (14)	−0.0023 (12)	0.0031 (11)	0.0025 (12)
O4	0.0205 (14)	0.0414 (19)	0.0256 (15)	0.0007 (13)	0.0006 (12)	0.0036 (14)
O5	0.0218 (15)	0.0393 (18)	0.0310 (16)	−0.0061 (14)	0.0015 (13)	0.0006 (14)
O6	0.0194 (14)	0.0391 (18)	0.0168 (13)	0.0036 (13)	0.0050 (11)	0.0013 (12)
O7	0.0216 (14)	0.0355 (17)	0.0163 (13)	0.0012 (12)	0.0060 (11)	−0.0006 (12)
O8	0.0287 (16)	0.0378 (18)	0.0178 (14)	0.0059 (13)	0.0054 (12)	−0.0004 (13)
O9	0.0215 (14)	0.0433 (19)	0.0169 (14)	0.0033 (13)	0.0012 (11)	0.0011 (13)
C1	0.021 (2)	0.038 (2)	0.0150 (18)	0.0019 (18)	0.0076 (16)	−0.0012 (17)
C2	0.0187 (19)	0.042 (3)	0.0176 (19)	0.0016 (18)	0.0066 (15)	−0.0012 (18)
C3	0.022 (2)	0.028 (2)	0.0195 (19)	−0.0008 (17)	0.0059 (15)	−0.0004 (17)
C4	0.0182 (18)	0.028 (2)	0.0199 (19)	0.0029 (17)	0.0043 (15)	0.0018 (16)
O10	0.0274 (17)	0.042 (2)	0.0349 (17)	−0.0011 (15)	0.0096 (14)	0.0058 (16)
O11	0.0386 (19)	0.046 (2)	0.0235 (16)	−0.0009 (17)	0.0009 (14)	0.0064 (15)

### Geometric parameters (Å, °)

**Table:**

Cr1—O2	1.965 (3)	O2—C1	1.286 (5)
Cr1—O1	1.965 (3)	O2—Na1^i^	2.591 (3)
Cr1—O1^i^	1.966 (3)	O3—C2	1.292 (5)
Cr1—O3	1.976 (3)	O4—C1	1.231 (5)
Cr1—O7	1.979 (3)	O4—Na2^v^	2.368 (4)
Cr1—O6	1.985 (3)	O5—C2	1.230 (5)
Cr1—Cr1^i^	2.9781 (13)	O5—Na2^v^	2.400 (4)
Na1—O8^ii^	2.368 (3)	O6—C4	1.288 (5)
Na1—O10	2.406 (4)	O6—Na2^vi^	2.418 (3)
Na1—O8^iii^	2.423 (3)	O7—C3	1.289 (5)
Na1—O9^iii^	2.452 (3)	O8—C4	1.219 (5)
Na1—O1	2.492 (3)	O8—Na1^vi^	2.368 (3)
Na1—O7	2.505 (3)	O8—Na1^vii^	2.423 (3)
Na1—O2^i^	2.591 (3)	O9—C3	1.220 (5)
Na1—C3^iii^	3.114 (4)	O9—Na1^vii^	2.452 (3)
Na1—C4^iii^	3.129 (4)	C1—C2	1.547 (7)
Na2—O9	2.359 (4)	C1—Na2^v^	3.067 (5)
Na2—O4^iv^	2.368 (4)	C2—Na2^v^	3.086 (4)
Na2—O10	2.389 (4)	C3—C4	1.556 (6)
Na2—O5^iv^	2.400 (4)	C3—Na1^vii^	3.114 (4)
Na2—O6^ii^	2.418 (3)	C4—Na1^vii^	3.129 (4)
Na2—C1^iv^	3.067 (5)	O10—H10A	0.905 (19)
Na2—C2^iv^	3.086 (4)	O10—H10B	0.88 (4)
Na2—C3	3.127 (5)	O11—H11A	0.88 (4)
O1—Cr1^i^	1.966 (3)	O11—H11B	0.879 (19)
O1—H1	0.8500		
			
O2—Cr1—O1	94.76 (12)	O9—Na2—O4^iv^	139.26 (13)
O2—Cr1—O1^i^	89.36 (12)	O9—Na2—O10	124.12 (13)
O1—Cr1—O1^i^	81.50 (13)	O4^iv^—Na2—O10	96.07 (13)
O2—Cr1—O3	82.37 (12)	O9—Na2—O5^iv^	87.29 (12)
O1—Cr1—O3	92.00 (12)	O4^iv^—Na2—O5^iv^	71.87 (12)
O1^i^—Cr1—O3	169.05 (12)	O10—Na2—O5^iv^	126.18 (13)
O2—Cr1—O7	175.76 (12)	O9—Na2—O6^ii^	77.65 (12)
O1—Cr1—O7	89.46 (12)	O4^iv^—Na2—O6^ii^	90.80 (12)
O1^i^—Cr1—O7	90.79 (12)	O10—Na2—O6^ii^	99.01 (12)
O3—Cr1—O7	97.99 (13)	O5^iv^—Na2—O6^ii^	132.29 (13)
O2—Cr1—O6	93.73 (12)	O9—Na2—C1^iv^	129.40 (12)
O1—Cr1—O6	170.95 (12)	O4^iv^—Na2—C1^iv^	21.68 (12)
O1^i^—Cr1—O6	95.49 (12)	O10—Na2—C1^iv^	104.93 (13)
O3—Cr1—O6	92.21 (13)	O5^iv^—Na2—C1^iv^	50.62 (12)
O7—Cr1—O6	82.03 (12)	O6^ii^—Na2—C1^iv^	108.42 (13)
O2—Cr1—Cr1^i^	92.71 (9)	O9—Na2—C2^iv^	106.68 (12)
O1—Cr1—Cr1^i^	40.76 (9)	O4^iv^—Na2—C2^iv^	50.63 (12)
O1^i^—Cr1—Cr1^i^	40.74 (8)	O10—Na2—C2^iv^	118.00 (13)
O3—Cr1—Cr1^i^	132.15 (9)	O5^iv^—Na2—C2^iv^	21.61 (12)
O7—Cr1—Cr1^i^	90.16 (9)	O6^ii^—Na2—C2^iv^	126.49 (13)
O6—Cr1—Cr1^i^	135.64 (10)	C1^iv^—Na2—C2^iv^	29.13 (12)
O2—Cr1—Na1	138.28 (9)	O9—Na2—C3	20.12 (10)
O1—Cr1—Na1	45.45 (9)	O4^iv^—Na2—C3	153.25 (13)
O1^i^—Cr1—Na1	94.96 (9)	O10—Na2—C3	105.60 (12)
O3—Cr1—Na1	86.57 (9)	O5^iv^—Na2—C3	105.94 (12)
O7—Cr1—Na1	45.92 (9)	O6^ii^—Na2—C3	70.73 (12)
O6—Cr1—Na1	126.90 (9)	C1^iv^—Na2—C3	149.17 (12)
Cr1^i^—Cr1—Na1	66.06 (4)	C2^iv^—Na2—C3	126.28 (13)
O2—Cr1—Na1^i^	46.42 (9)	O9—Na2—Na1	85.31 (9)
O1—Cr1—Na1^i^	92.56 (10)	O4^iv^—Na2—Na1	129.94 (10)
O1^i^—Cr1—Na1^i^	43.47 (9)	O10—Na2—Na1	41.66 (9)
O3—Cr1—Na1^i^	128.78 (9)	O5^iv^—Na2—Na1	148.19 (11)
O7—Cr1—Na1^i^	133.03 (9)	O6^ii^—Na2—Na1	75.87 (8)
O6—Cr1—Na1^i^	91.04 (10)	C1^iv^—Na2—Na1	145.29 (10)
Cr1^i^—Cr1—Na1^i^	63.28 (4)	C2^iv^—Na2—Na1	155.93 (10)
Na1—Cr1—Na1^i^	129.34 (3)	C3—Na2—Na1	65.47 (8)
O8^ii^—Na1—O10	88.06 (12)	O9—Na2—Na1^vii^	31.00 (8)
O8^ii^—Na1—O8^iii^	73.25 (12)	O4^iv^—Na2—Na1^vii^	108.28 (10)
O10—Na1—O8^iii^	133.10 (14)	O10—Na2—Na1^vii^	153.53 (10)
O8^ii^—Na1—O9^iii^	131.85 (13)	O5^iv^—Na2—Na1^vii^	72.98 (9)
O10—Na1—O9^iii^	95.89 (12)	O6^ii^—Na2—Na1^vii^	71.10 (9)
O8^iii^—Na1—O9^iii^	69.37 (11)	C1^iv^—Na2—Na1^vii^	101.51 (9)
O8^ii^—Na1—O1	118.75 (12)	C2^iv^—Na2—Na1^vii^	86.29 (9)
O10—Na1—O1	145.98 (14)	C3—Na2—Na1^vii^	48.18 (8)
O8^iii^—Na1—O1	77.96 (11)	Na1—Na2—Na1^vii^	112.08 (6)
O9^iii^—Na1—O1	81.77 (11)	Cr1—O1—Cr1^i^	98.50 (13)
O8^ii^—Na1—O7	77.46 (11)	Cr1—O1—Na1	100.35 (12)
O10—Na1—O7	101.98 (12)	Cr1^i^—O1—Na1	103.65 (12)
O8^iii^—Na1—O7	114.66 (12)	Cr1—O1—H1	121.6
O9^iii^—Na1—O7	146.39 (13)	Cr1^i^—O1—H1	122.4
O1—Na1—O7	67.50 (10)	Na1—O1—H1	107.1
O8^ii^—Na1—O2^i^	146.29 (12)	C1—O2—Cr1	114.7 (3)
O10—Na1—O2^i^	80.21 (12)	C1—O2—Na1^i^	144.1 (3)
O8^iii^—Na1—O2^i^	136.02 (12)	Cr1—O2—Na1^i^	100.25 (12)
O9^iii^—Na1—O2^i^	81.06 (11)	C2—O3—Cr1	113.8 (3)
O1—Na1—O2^i^	65.86 (10)	C1—O4—Na2^v^	113.0 (3)
O7—Na1—O2^i^	74.32 (10)	C2—O5—Na2^v^	112.4 (3)
O8^ii^—Na1—C3^iii^	119.34 (12)	C4—O6—Cr1	114.1 (2)
O10—Na1—C3^iii^	114.87 (12)	C4—O6—Na2^vi^	125.5 (2)
O8^iii^—Na1—C3^iii^	49.52 (11)	Cr1—O6—Na2^vi^	119.51 (13)
O9^iii^—Na1—C3^iii^	21.37 (10)	C3—O7—Cr1	113.1 (3)
O1—Na1—C3^iii^	71.88 (11)	C3—O7—Na1	147.0 (3)
O7—Na1—C3^iii^	139.02 (12)	Cr1—O7—Na1	99.50 (11)
O2^i^—Na1—C3^iii^	94.18 (11)	C4—O8—Na1^vi^	135.9 (3)
O8^ii^—Na1—C4^iii^	93.25 (12)	C4—O8—Na1^vii^	114.3 (3)
O10—Na1—C4^iii^	130.64 (13)	Na1^vi^—O8—Na1^vii^	106.75 (12)
O8^iii^—Na1—C4^iii^	20.80 (10)	C3—O9—Na2	118.2 (3)
O9^iii^—Na1—C4^iii^	49.37 (10)	C3—O9—Na1^vii^	111.6 (3)
O1—Na1—C4^iii^	72.13 (11)	Na2—O9—Na1^vii^	119.28 (14)
O7—Na1—C4^iii^	126.47 (11)	O4—C1—O2	125.4 (4)
O2^i^—Na1—C4^iii^	118.33 (11)	O4—C1—C2	120.8 (4)
C3^iii^—Na1—C4^iii^	28.86 (10)	O2—C1—C2	113.8 (3)
O8^ii^—Na1—Cr1	93.80 (9)	O4—C1—Na2^v^	45.3 (2)
O10—Na1—Cr1	133.43 (11)	O2—C1—Na2^v^	168.2 (3)
O8^iii^—Na1—Cr1	91.29 (9)	C2—C1—Na2^v^	76.1 (2)
O9^iii^—Na1—Cr1	115.97 (10)	O5—C2—O3	125.5 (4)
O1—Na1—Cr1	34.20 (7)	O5—C2—C1	120.3 (4)
O7—Na1—Cr1	34.58 (7)	O3—C2—C1	114.1 (4)
O2^i^—Na1—Cr1	72.85 (8)	O5—C2—Na2^v^	46.0 (2)
C3^iii^—Na1—Cr1	104.50 (9)	O3—C2—Na2^v^	169.9 (3)
C4^iii^—Na1—Cr1	95.76 (9)	C1—C2—Na2^v^	74.8 (2)
O8^ii^—Na1—Cr1^i^	144.36 (10)	O9—C3—O7	126.2 (4)
O10—Na1—Cr1^i^	113.46 (10)	O9—C3—C4	120.1 (4)
O8^iii^—Na1—Cr1^i^	105.90 (9)	O7—C3—C4	113.7 (3)
O9^iii^—Na1—Cr1^i^	76.17 (9)	O9—C3—Na1^vii^	47.1 (2)
O1—Na1—Cr1^i^	32.88 (7)	O7—C3—Na1^vii^	161.1 (3)
O7—Na1—Cr1^i^	70.66 (8)	C4—C3—Na1^vii^	76.1 (2)
O2^i^—Na1—Cr1^i^	33.33 (7)	O7—C3—Na2	85.3 (2)
C3^iii^—Na1—Cr1^i^	78.41 (9)	C4—C3—Na2	159.3 (3)
C4^iii^—Na1—Cr1^i^	93.08 (9)	Na1^vii^—C3—Na2	83.39 (11)
Cr1—Na1—Cr1^i^	50.66 (3)	O8—C4—O6	126.7 (4)
O8^ii^—Na1—Na2	70.09 (9)	O8—C4—C3	119.5 (4)
O10—Na1—Na2	41.28 (9)	O6—C4—C3	113.8 (3)
O8^iii^—Na1—Na2	142.94 (10)	O6—C4—Na1^vii^	169.2 (3)
O9^iii^—Na1—Na2	135.70 (9)	C3—C4—Na1^vii^	75.1 (2)
O1—Na1—Na2	125.14 (9)	Na2—O10—Na1	97.06 (13)
O7—Na1—Na2	62.18 (8)	Na2—O10—H10A	114 (3)
O2^i^—Na1—Na2	80.56 (8)	Na1—O10—H10A	116 (4)
C3^iii^—Na1—Na2	156.03 (10)	Na2—O10—H10B	114 (3)
C4^iii^—Na1—Na2	160.00 (10)	Na1—O10—H10B	109 (3)
Cr1—Na1—Na2	96.31 (5)	H10A—O10—H10B	106 (3)
Cr1^i^—Na1—Na2	106.90 (6)	H11A—O11—H11B	112 (3)
			
O2—Cr1—Na1—O8^ii^	117.77 (15)	O8^ii^—Na1—O1—Cr1^i^	−149.18 (12)
O1—Cr1—Na1—O8^ii^	139.46 (15)	O10—Na1—O1—Cr1^i^	−11.1 (3)
O1^i^—Cr1—Na1—O8^ii^	−147.61 (12)	O8^iii^—Na1—O1—Cr1^i^	147.75 (13)
O3—Cr1—Na1—O8^ii^	43.26 (12)	O9^iii^—Na1—O1—Cr1^i^	77.21 (12)
O7—Cr1—Na1—O8^ii^	−61.33 (14)	O7—Na1—O1—Cr1^i^	−88.88 (13)
O6—Cr1—Na1—O8^ii^	−46.93 (15)	O2^i^—Na1—O1—Cr1^i^	−6.63 (10)
Cr1^i^—Cr1—Na1—O8^ii^	−177.08 (9)	C3^iii^—Na1—O1—Cr1^i^	96.70 (13)
Na1^i^—Cr1—Na1—O8^ii^	−177.08 (9)	C4^iii^—Na1—O1—Cr1^i^	127.07 (13)
O2—Cr1—Na1—O10	−151.31 (18)	Cr1—Na1—O1—Cr1^i^	−101.47 (15)
O1—Cr1—Na1—O10	−129.62 (19)	Na2—Na1—O1—Cr1^i^	−64.28 (14)
O1^i^—Cr1—Na1—O10	−56.69 (16)	O1—Cr1—O2—C1	−82.6 (3)
O3—Cr1—Na1—O10	134.18 (16)	O1^i^—Cr1—O2—C1	−164.0 (3)
O7—Cr1—Na1—O10	29.59 (17)	O3—Cr1—O2—C1	8.8 (3)
O6—Cr1—Na1—O10	43.99 (19)	O6—Cr1—O2—C1	100.5 (3)
Cr1^i^—Cr1—Na1—O10	−86.16 (14)	Cr1^i^—Cr1—O2—C1	−123.4 (3)
Na1^i^—Cr1—Na1—O10	−86.16 (14)	Na1—Cr1—O2—C1	−67.3 (3)
O2—Cr1—Na1—O8^iii^	44.47 (16)	Na1^i^—Cr1—O2—C1	−171.6 (3)
O1—Cr1—Na1—O8^iii^	66.16 (14)	O1—Cr1—O2—Na1^i^	89.00 (12)
O1^i^—Cr1—Na1—O8^iii^	139.09 (11)	O1^i^—Cr1—O2—Na1^i^	7.58 (12)
O3—Cr1—Na1—O8^iii^	−30.04 (12)	O3—Cr1—O2—Na1^i^	−179.61 (12)
O7—Cr1—Na1—O8^iii^	−134.63 (15)	O6—Cr1—O2—Na1^i^	−87.88 (13)
O6—Cr1—Na1—O8^iii^	−120.23 (14)	Cr1^i^—Cr1—O2—Na1^i^	48.20 (9)
Cr1^i^—Cr1—Na1—O8^iii^	109.62 (9)	Na1—Cr1—O2—Na1^i^	104.33 (12)
Na1^i^—Cr1—Na1—O8^iii^	109.62 (9)	O2—Cr1—O3—C2	−9.6 (3)
O2—Cr1—Na1—O9^iii^	−23.14 (18)	O1—Cr1—O3—C2	84.9 (3)
O1—Cr1—Na1—O9^iii^	−1.45 (14)	O1^i^—Cr1—O3—C2	31.6 (8)
O1^i^—Cr1—Na1—O9^iii^	71.48 (13)	O7—Cr1—O3—C2	174.6 (3)
O3—Cr1—Na1—O9^iii^	−97.65 (13)	O6—Cr1—O3—C2	−103.1 (3)
O7—Cr1—Na1—O9^iii^	157.76 (17)	Cr1^i^—Cr1—O3—C2	77.1 (3)
O6—Cr1—Na1—O9^iii^	172.16 (14)	Na1—Cr1—O3—C2	130.1 (3)
Cr1^i^—Cr1—Na1—O9^iii^	42.01 (10)	Na1^i^—Cr1—O3—C2	−10.0 (3)
Na1^i^—Cr1—Na1—O9^iii^	42.01 (10)	O2—Cr1—O6—C4	168.9 (3)
O2—Cr1—Na1—O1	−21.69 (18)	O1^i^—Cr1—O6—C4	79.2 (3)
O1^i^—Cr1—Na1—O1	72.93 (16)	O3—Cr1—O6—C4	−108.6 (3)
O3—Cr1—Na1—O1	−96.20 (15)	O7—Cr1—O6—C4	−10.9 (3)
O7—Cr1—Na1—O1	159.21 (18)	Cr1^i^—Cr1—O6—C4	71.2 (3)
O6—Cr1—Na1—O1	173.61 (17)	Na1—Cr1—O6—C4	−21.2 (3)
Cr1^i^—Cr1—Na1—O1	43.46 (12)	Na1^i^—Cr1—O6—C4	122.5 (3)
Na1^i^—Cr1—Na1—O1	43.46 (12)	O2—Cr1—O6—Na2^vi^	−0.77 (18)
O2—Cr1—Na1—O7	179.10 (18)	O1^i^—Cr1—O6—Na2^vi^	−90.48 (17)
O1—Cr1—Na1—O7	−159.21 (18)	O3—Cr1—O6—Na2^vi^	81.72 (17)
O1^i^—Cr1—Na1—O7	−86.29 (15)	O7—Cr1—O6—Na2^vi^	179.49 (18)
O3—Cr1—Na1—O7	104.59 (15)	Cr1^i^—Cr1—O6—Na2^vi^	−98.50 (16)
O6—Cr1—Na1—O7	14.40 (17)	Na1—Cr1—O6—Na2^vi^	169.10 (10)
Cr1^i^—Cr1—Na1—O7	−115.75 (13)	Na1^i^—Cr1—O6—Na2^vi^	−47.15 (15)
Na1^i^—Cr1—Na1—O7	−115.75 (13)	O1—Cr1—O7—C3	−159.9 (3)
O2—Cr1—Na1—O2^i^	−93.80 (14)	O1^i^—Cr1—O7—C3	−78.4 (3)
O1—Cr1—Na1—O2^i^	−72.11 (14)	O3—Cr1—O7—C3	108.1 (3)
O1^i^—Cr1—Na1—O2^i^	0.82 (11)	O6—Cr1—O7—C3	17.0 (3)
O3—Cr1—Na1—O2^i^	−168.31 (11)	Cr1^i^—Cr1—O7—C3	−119.2 (3)
O7—Cr1—Na1—O2^i^	87.10 (14)	Na1—Cr1—O7—C3	−174.6 (3)
O6—Cr1—Na1—O2^i^	101.50 (14)	Na1^i^—Cr1—O7—C3	−66.9 (3)
Cr1^i^—Cr1—Na1—O2^i^	−28.65 (7)	O1—Cr1—O7—Na1	14.65 (13)
Na1^i^—Cr1—Na1—O2^i^	−28.65 (7)	O1^i^—Cr1—O7—Na1	96.15 (12)
O2—Cr1—Na1—C3^iii^	−3.86 (17)	O3—Cr1—O7—Na1	−77.29 (13)
O1—Cr1—Na1—C3^iii^	17.83 (14)	O6—Cr1—O7—Na1	−168.42 (14)
O1^i^—Cr1—Na1—C3^iii^	90.76 (12)	Cr1^i^—Cr1—O7—Na1	55.41 (10)
O3—Cr1—Na1—C3^iii^	−78.37 (12)	Na1^i^—Cr1—O7—Na1	107.64 (11)
O7—Cr1—Na1—C3^iii^	177.04 (16)	O8^ii^—Na1—O7—C3	−72.9 (5)
O6—Cr1—Na1—C3^iii^	−168.56 (14)	O10—Na1—O7—C3	12.3 (5)
Cr1^i^—Cr1—Na1—C3^iii^	61.29 (9)	O8^iii^—Na1—O7—C3	−137.7 (5)
Na1^i^—Cr1—Na1—C3^iii^	61.29 (9)	O9^iii^—Na1—O7—C3	132.9 (5)
O2—Cr1—Na1—C4^iii^	24.11 (16)	O1—Na1—O7—C3	158.3 (5)
O1—Cr1—Na1—C4^iii^	45.80 (14)	O2^i^—Na1—O7—C3	88.4 (5)
O1^i^—Cr1—Na1—C4^iii^	118.73 (11)	C3^iii^—Na1—O7—C3	166.4 (4)
O3—Cr1—Na1—C4^iii^	−50.39 (12)	C4^iii^—Na1—O7—C3	−157.6 (5)
O7—Cr1—Na1—C4^iii^	−154.98 (15)	Cr1—Na1—O7—C3	170.8 (6)
O6—Cr1—Na1—C4^iii^	−140.59 (14)	Cr1^i^—Na1—O7—C3	123.2 (5)
Cr1^i^—Cr1—Na1—C4^iii^	89.26 (9)	Na2—Na1—O7—C3	1.0 (5)
Na1^i^—Cr1—Na1—C4^iii^	89.27 (9)	O8^ii^—Na1—O7—Cr1	116.25 (13)
O2—Cr1—Na1—Cr1^i^	−65.15 (13)	O10—Na1—O7—Cr1	−158.49 (13)
O1—Cr1—Na1—Cr1^i^	−43.46 (12)	O8^iii^—Na1—O7—Cr1	51.52 (16)
O1^i^—Cr1—Na1—Cr1^i^	29.47 (8)	O9^iii^—Na1—O7—Cr1	−37.9 (3)
O3—Cr1—Na1—Cr1^i^	−139.66 (9)	O1—Na1—O7—Cr1	−12.47 (11)
O7—Cr1—Na1—Cr1^i^	115.75 (13)	O2^i^—Na1—O7—Cr1	−82.38 (12)
O6—Cr1—Na1—Cr1^i^	130.15 (12)	C3^iii^—Na1—O7—Cr1	−4.4 (2)
Na1^i^—Cr1—Na1—Cr1^i^	0.0	C4^iii^—Na1—O7—Cr1	31.54 (19)
O2—Cr1—Na1—Na2	−171.87 (13)	Cr1^i^—Na1—O7—Cr1	−47.58 (9)
O1—Cr1—Na1—Na2	−150.17 (14)	Na2—Na1—O7—Cr1	−169.83 (14)
O1^i^—Cr1—Na1—Na2	−77.25 (9)	O4^iv^—Na2—O9—C3	144.0 (3)
O3—Cr1—Na1—Na2	113.63 (9)	O10—Na2—O9—C3	−25.2 (4)
O7—Cr1—Na1—Na2	9.04 (12)	O5^iv^—Na2—O9—C3	−158.2 (3)
O6—Cr1—Na1—Na2	23.44 (13)	O6^ii^—Na2—O9—C3	67.4 (3)
Cr1^i^—Cr1—Na1—Na2	−106.71 (5)	C1^iv^—Na2—O9—C3	171.2 (3)
Na1^i^—Cr1—Na1—Na2	−106.71 (5)	C2^iv^—Na2—O9—C3	−167.9 (3)
O8^ii^—Na1—Na2—O9	89.19 (13)	Na1—Na2—O9—C3	−9.2 (3)
O10—Na1—Na2—O9	−159.84 (17)	Na1^vii^—Na2—O9—C3	141.1 (4)
O8^iii^—Na1—Na2—O9	98.03 (19)	O4^iv^—Na2—O9—Na1^vii^	2.8 (3)
O9^iii^—Na1—Na2—O9	−140.65 (12)	O10—Na2—O9—Na1^vii^	−166.37 (15)
O1—Na1—Na2—O9	−22.57 (14)	O5^iv^—Na2—O9—Na1^vii^	60.65 (17)
O7—Na1—Na2—O9	3.21 (12)	O6^ii^—Na2—O9—Na1^vii^	−73.77 (16)
O2^i^—Na1—Na2—O9	−73.96 (11)	C1^iv^—Na2—O9—Na1^vii^	30.1 (2)
C3^iii^—Na1—Na2—O9	−152.9 (3)	C2^iv^—Na2—O9—Na1^vii^	50.98 (19)
C4^iii^—Na1—Na2—O9	124.2 (3)	C3—Na2—O9—Na1^vii^	−141.1 (4)
Cr1—Na1—Na2—O9	−2.58 (10)	Na1—Na2—O9—Na1^vii^	−150.31 (14)
Cr1^i^—Na1—Na2—O9	−53.31 (10)	Na2^v^—O4—C1—O2	−170.5 (3)
O8^ii^—Na1—Na2—O4^iv^	−68.17 (16)	Na2^v^—O4—C1—C2	10.5 (4)
O10—Na1—Na2—O4^iv^	42.79 (18)	Cr1—O2—C1—O4	174.5 (3)
O8^iii^—Na1—Na2—O4^iv^	−59.3 (2)	Na1^i^—O2—C1—O4	8.7 (7)
O9^iii^—Na1—Na2—O4^iv^	62.0 (2)	Cr1—O2—C1—C2	−6.5 (4)
O1—Na1—Na2—O4^iv^	−179.93 (15)	Na1^i^—O2—C1—C2	−172.3 (3)
O7—Na1—Na2—O4^iv^	−154.15 (16)	Cr1—O2—C1—Na2^v^	139.7 (13)
O2^i^—Na1—Na2—O4^iv^	128.68 (15)	Na1^i^—O2—C1—Na2^v^	−26.1 (17)
C3^iii^—Na1—Na2—O4^iv^	49.8 (3)	Na2^v^—O5—C2—O3	172.8 (3)
C4^iii^—Na1—Na2—O4^iv^	−33.1 (3)	Na2^v^—O5—C2—C1	−8.3 (4)
Cr1—Na1—Na2—O4^iv^	−159.94 (13)	Cr1—O3—C2—O5	−172.4 (3)
Cr1^i^—Na1—Na2—O4^iv^	149.33 (13)	Cr1—O3—C2—C1	8.7 (4)
O8^ii^—Na1—Na2—O10	−110.96 (17)	Cr1—O3—C2—Na2^v^	−141.6 (15)
O8^iii^—Na1—Na2—O10	−102.1 (2)	O4—C1—C2—O5	−1.5 (6)
O9^iii^—Na1—Na2—O10	19.20 (19)	O2—C1—C2—O5	179.5 (4)
O1—Na1—Na2—O10	137.27 (18)	Na2^v^—C1—C2—O5	6.2 (3)
O7—Na1—Na2—O10	163.05 (17)	O4—C1—C2—O3	177.5 (4)
O2^i^—Na1—Na2—O10	85.88 (15)	O2—C1—C2—O3	−1.6 (5)
C3^iii^—Na1—Na2—O10	7.0 (3)	Na2^v^—C1—C2—O3	−174.8 (3)
C4^iii^—Na1—Na2—O10	−75.9 (3)	O4—C1—C2—Na2^v^	−7.7 (3)
Cr1—Na1—Na2—O10	157.27 (15)	O2—C1—C2—Na2^v^	173.3 (3)
Cr1^i^—Na1—Na2—O10	106.54 (14)	Na2—O9—C3—O7	13.0 (6)
O8^ii^—Na1—Na2—O5^iv^	166.4 (2)	Na1^vii^—O9—C3—O7	156.9 (4)
O10—Na1—Na2—O5^iv^	−82.7 (2)	Na2—O9—C3—C4	−167.3 (3)
O8^iii^—Na1—Na2—O5^iv^	175.2 (2)	Na1^vii^—O9—C3—C4	−23.4 (5)
O9^iii^—Na1—Na2—O5^iv^	−63.5 (3)	Na2—O9—C3—Na1^vii^	−144.0 (4)
O1—Na1—Na2—O5^iv^	54.6 (2)	Na1^vii^—O9—C3—Na2	144.0 (4)
O7—Na1—Na2—O5^iv^	80.4 (2)	Cr1—O7—C3—O9	160.5 (4)
O2^i^—Na1—Na2—O5^iv^	3.2 (2)	Na1—O7—C3—O9	−9.6 (8)
C3^iii^—Na1—Na2—O5^iv^	−75.7 (3)	Cr1—O7—C3—C4	−19.2 (4)
C4^iii^—Na1—Na2—O5^iv^	−158.6 (3)	Na1—O7—C3—C4	170.7 (3)
Cr1—Na1—Na2—O5^iv^	74.6 (2)	Cr1—O7—C3—Na1^vii^	−137.3 (8)
Cr1^i^—Na1—Na2—O5^iv^	23.9 (2)	Na1—O7—C3—Na1^vii^	52.6 (12)
O8^ii^—Na1—Na2—O6^ii^	10.76 (12)	Cr1—O7—C3—Na2	169.14 (17)
O10—Na1—Na2—O6^ii^	121.73 (17)	Na1—O7—C3—Na2	−1.0 (5)
O8^iii^—Na1—Na2—O6^ii^	19.60 (19)	O4^iv^—Na2—C3—O9	−58.6 (5)
O9^iii^—Na1—Na2—O6^ii^	140.92 (17)	O10—Na2—C3—O9	158.5 (3)
O1—Na1—Na2—O6^ii^	−101.00 (13)	O5^iv^—Na2—C3—O9	22.7 (3)
O7—Na1—Na2—O6^ii^	−75.22 (12)	O6^ii^—Na2—C3—O9	−107.2 (3)
O2^i^—Na1—Na2—O6^ii^	−152.39 (11)	C1^iv^—Na2—C3—O9	−13.3 (4)
C3^iii^—Na1—Na2—O6^ii^	128.7 (3)	C2^iv^—Na2—C3—O9	14.5 (4)
C4^iii^—Na1—Na2—O6^ii^	45.8 (3)	Na1—Na2—C3—O9	170.0 (3)
Cr1—Na1—Na2—O6^ii^	−81.01 (9)	Na1^vii^—Na2—C3—O9	−25.7 (3)
Cr1^i^—Na1—Na2—O6^ii^	−131.74 (9)	O9—Na2—C3—O7	−169.5 (5)
O8^ii^—Na1—Na2—C1^iv^	−91.3 (2)	O4^iv^—Na2—C3—O7	131.9 (3)
O10—Na1—Na2—C1^iv^	19.7 (2)	O10—Na2—C3—O7	−11.0 (3)
O8^iii^—Na1—Na2—C1^iv^	−82.5 (2)	O5^iv^—Na2—C3—O7	−146.8 (2)
O9^iii^—Na1—Na2—C1^iv^	38.9 (3)	O6^ii^—Na2—C3—O7	83.3 (2)
O1—Na1—Na2—C1^iv^	156.94 (19)	C1^iv^—Na2—C3—O7	177.1 (3)
O7—Na1—Na2—C1^iv^	−177.3 (2)	C2^iv^—Na2—C3—O7	−155.1 (2)
O2^i^—Na1—Na2—C1^iv^	105.5 (2)	Na1—Na2—C3—O7	0.4 (2)
C3^iii^—Na1—Na2—C1^iv^	26.6 (3)	Na1^vii^—Na2—C3—O7	164.8 (3)
C4^iii^—Na1—Na2—C1^iv^	−56.3 (3)	O9—Na2—C3—C4	32.6 (7)
Cr1—Na1—Na2—C1^iv^	176.93 (18)	O4^iv^—Na2—C3—C4	−26.0 (10)
Cr1^i^—Na1—Na2—C1^iv^	126.20 (18)	O10—Na2—C3—C4	−168.9 (8)
O8^ii^—Na1—Na2—C2^iv^	−149.3 (3)	O5^iv^—Na2—C3—C4	55.3 (8)
O10—Na1—Na2—C2^iv^	−38.4 (3)	O6^ii^—Na2—C3—C4	−74.6 (8)
O8^iii^—Na1—Na2—C2^iv^	−140.5 (3)	C1^iv^—Na2—C3—C4	19.2 (9)
O9^iii^—Na1—Na2—C2^iv^	−19.2 (3)	C2^iv^—Na2—C3—C4	47.0 (8)
O1—Na1—Na2—C2^iv^	98.9 (3)	Na1—Na2—C3—C4	−157.5 (8)
O7—Na1—Na2—C2^iv^	124.7 (3)	Na1^vii^—Na2—C3—C4	6.9 (7)
O2^i^—Na1—Na2—C2^iv^	47.5 (3)	O9—Na2—C3—Na1^vii^	25.7 (3)
C3^iii^—Na1—Na2—C2^iv^	−31.4 (4)	O4^iv^—Na2—C3—Na1^vii^	−32.9 (3)
C4^iii^—Na1—Na2—C2^iv^	−114.3 (4)	O10—Na2—C3—Na1^vii^	−175.79 (12)
Cr1—Na1—Na2—C2^iv^	118.9 (3)	O5^iv^—Na2—C3—Na1^vii^	48.39 (13)
Cr1^i^—Na1—Na2—C2^iv^	68.2 (3)	O6^ii^—Na2—C3—Na1^vii^	−81.51 (11)
O8^ii^—Na1—Na2—C3	85.74 (12)	C1^iv^—Na2—C3—Na1^vii^	12.4 (3)
O10—Na1—Na2—C3	−163.30 (17)	C2^iv^—Na2—C3—Na1^vii^	40.15 (17)
O8^iii^—Na1—Na2—C3	94.57 (19)	Na1—Na2—C3—Na1^vii^	−164.35 (11)
O9^iii^—Na1—Na2—C3	−144.10 (18)	Na1^vi^—O8—C4—O6	32.1 (7)
O1—Na1—Na2—C3	−26.02 (13)	Na1^vii^—O8—C4—O6	−170.9 (4)
O7—Na1—Na2—C3	−0.24 (12)	Na1^vi^—O8—C4—C3	−148.0 (3)
O2^i^—Na1—Na2—C3	−77.41 (11)	Na1^vii^—O8—C4—C3	9.0 (5)
C3^iii^—Na1—Na2—C3	−156.3 (2)	Na1^vi^—O8—C4—Na1^vii^	−157.0 (5)
C4^iii^—Na1—Na2—C3	120.8 (3)	Cr1—O6—C4—O8	−176.3 (4)
Cr1—Na1—Na2—C3	−6.03 (9)	Na2^vi^—O6—C4—O8	−7.4 (6)
Cr1^i^—Na1—Na2—C3	−56.76 (10)	Cr1—O6—C4—C3	3.7 (4)
O8^ii^—Na1—Na2—Na1^vii^	73.21 (9)	Na2^vi^—O6—C4—C3	172.7 (3)
O10—Na1—Na2—Na1^vii^	−175.83 (16)	Cr1—O6—C4—Na1^vii^	147.1 (15)
O8^iii^—Na1—Na2—Na1^vii^	82.04 (16)	Na2^vi^—O6—C4—Na1^vii^	−43.9 (18)
O9^iii^—Na1—Na2—Na1^vii^	−156.63 (15)	O9—C3—C4—O8	10.8 (6)
O1—Na1—Na2—Na1^vii^	−38.55 (12)	O7—C3—C4—O8	−169.4 (4)
O7—Na1—Na2—Na1^vii^	−12.77 (9)	Na1^vii^—C3—C4—O8	−6.6 (4)
O2^i^—Na1—Na2—Na1^vii^	−89.94 (9)	Na2—C3—C4—O8	−13.6 (10)
C3^iii^—Na1—Na2—Na1^vii^	−168.9 (3)	O9—C3—C4—O6	−169.3 (4)
C4^iii^—Na1—Na2—Na1^vii^	108.2 (3)	O7—C3—C4—O6	10.5 (5)
Cr1—Na1—Na2—Na1^vii^	−18.56 (6)	Na1^vii^—C3—C4—O6	173.3 (4)
Cr1^i^—Na1—Na2—Na1^vii^	−69.29 (7)	Na2—C3—C4—O6	166.3 (6)
O2—Cr1—O1—Cr1^i^	−88.64 (13)	O9—C3—C4—Na1^vii^	17.4 (4)
O1^i^—Cr1—O1—Cr1^i^	0.0	O7—C3—C4—Na1^vii^	−162.9 (4)
O3—Cr1—O1—Cr1^i^	−171.14 (13)	Na2—C3—C4—Na1^vii^	−7.0 (8)
O7—Cr1—O1—Cr1^i^	90.88 (13)	O9—Na2—O10—Na1	24.5 (2)
Na1—Cr1—O1—Cr1^i^	105.65 (14)	O4^iv^—Na2—O10—Na1	−148.41 (13)
Na1^i^—Cr1—O1—Cr1^i^	−42.17 (10)	O5^iv^—Na2—O10—Na1	139.64 (15)
O2—Cr1—O1—Na1	165.71 (12)	O6^ii^—Na2—O10—Na1	−56.63 (15)
O1^i^—Cr1—O1—Na1	−105.65 (14)	C1^iv^—Na2—O10—Na1	−168.56 (13)
O3—Cr1—O1—Na1	83.21 (13)	C2^iv^—Na2—O10—Na1	163.34 (14)
O7—Cr1—O1—Na1	−14.77 (13)	C3—Na2—O10—Na1	15.75 (16)
Cr1^i^—Cr1—O1—Na1	−105.65 (14)	Na1^vii^—Na2—O10—Na1	8.7 (3)
Na1^i^—Cr1—O1—Na1	−147.82 (8)	O8^ii^—Na1—O10—Na2	61.46 (14)
O8^ii^—Na1—O1—Cr1	−47.71 (16)	O8^iii^—Na1—O10—Na2	126.21 (16)
O10—Na1—O1—Cr1	90.3 (2)	O9^iii^—Na1—O10—Na2	−166.65 (13)
O8^iii^—Na1—O1—Cr1	−110.77 (13)	O1—Na1—O10—Na2	−82.6 (2)
O9^iii^—Na1—O1—Cr1	178.69 (13)	O7—Na1—O10—Na2	−15.28 (15)
O7—Na1—O1—Cr1	12.59 (11)	O2^i^—Na1—O10—Na2	−86.80 (13)
O2^i^—Na1—O1—Cr1	94.84 (13)	C3^iii^—Na1—O10—Na2	−176.89 (13)
C3^iii^—Na1—O1—Cr1	−161.83 (14)	C4^iii^—Na1—O10—Na2	154.08 (14)
C4^iii^—Na1—O1—Cr1	−131.45 (14)	Cr1—Na1—O10—Na2	−31.93 (19)
Cr1^i^—Na1—O1—Cr1	101.47 (15)	Cr1^i^—Na1—O10—Na2	−89.20 (12)
Na2—Na1—O1—Cr1	37.20 (16)		

Symmetry codes: (i) −*x*+1, −*y*, −*z*+1; (ii) −*x*+1, *y*+1/2, −*z*+1/2; (iii) *x*, −*y*+1/2, *z*+1/2; (iv) *x*−1, −*y*+1/2, *z*−1/2; (v) *x*+1, −*y*+1/2, *z*+1/2; (vi) −*x*+1, *y*−1/2, −*z*+1/2; (vii) *x*, −*y*+1/2, *z*−1/2.

### Hydrogen-bond geometry (Å, °)

**Table:**

*D*—H···*A*	*D*—H	H···*A*	*D*···*A*	*D*—H···*A*
O11—H11B···O9^ii^	0.88 (2)	2.23 (2)	3.080 (5)	163 (5)
O11—H11A···O6	0.88 (4)	2.53 (4)	3.144 (5)	128 (4)
O11—H11A···O3	0.88 (4)	2.25 (4)	2.949 (4)	137 (4)
O10—H10B···O5^viii^	0.88 (4)	2.36 (4)	3.123 (4)	144 (5)
O10—H10A···O5^ix^	0.91 (2)	2.02 (2)	2.922 (5)	179 (4)
O1—H1···O11^iii^	0.85	2.01	2.808 (4)	156

Symmetry codes: (ii) −*x*+1, *y*+1/2, −*z*+1/2; (viii) *x*−1, *y*, *z*; (ix) −*x*+1, −*y*+1, −*z*+1; (iii) *x*, −*y*+1/2, *z*+1/2.
